# Virtual and In Vitro Screening of Natural Products Identifies Indole and Benzene Derivatives as Inhibitors of SARS-CoV-2 Main Protease (M^pro^)

**DOI:** 10.3390/biology12040519

**Published:** 2023-03-29

**Authors:** Dony Ang, Riley Kendall, Hagop S. Atamian

**Affiliations:** 1 Computational and Data Sciences Program, Chapman University, Orange, CA 92866, USA; 2 Schmid College of Science and Technology, Chapman University, Orange, CA 92866, USA; 3 Biological Sciences Program, Chapman University, Orange, CA 92866, USA

**Keywords:** COVID-19, computation, structure-based virtual screening, protease inhibitor, ligand-based virtual screening, in vitro, drug-target interaction

## Abstract

**Simple Summary:**

The coronavirus disease 2019 (COVID-19) pandemic caused more than 6.7 million deaths worldwide. Certain groups of individuals are still at a high risk of severe illness. The availability of drugs to treat COVID-19 symptoms will save many lives. The main protease (M^pro^) of SARS-CoV2, the causal agent of COVID-19, is a promising target for drug discovery. Natural products have been used for thousands of years to treat diseases and represent valuable resources for drug discovery. While the process of experimentally screening chemicals for drug discovery has often been very long and expensive, recent advances in virtual screening have made it possible to screen millions of potential chemicals in a very short time using computers. In this research, around 400,000 natural products were virtually screened within a month and narrowed down to 20 products that could potentially bind to the SARS-CoV2 M^pro^. In vitro experimental testing of seven natural products demonstrated that the virtual screening approach used in this study had a significantly high rate of accuracy since more than 50% of the experimentally tested natural products (four out of seven) were able to inhibit the function of the M^pro^ in real-life consistent with the computer predictions. Our results show that with further research, beta-carboline, N-alkyl indole, and Benzoic acid ester types of natural products could be used in treating COVID-19 in the future.

**Abstract:**

The rapid spread of the coronavirus disease 2019 (COVID-19) resulted in serious health, social, and economic consequences. While the development of effective vaccines substantially reduced the severity of symptoms and the associated deaths, we still urgently need effective drugs to further reduce the number of casualties associated with SARS-CoV-2 infections. Machine learning methods both improved and sped up all the different stages of the drug discovery processes by performing complex analyses with enormous datasets. Natural products (NPs) have been used for treating diseases and infections for thousands of years and represent a valuable resource for drug discovery when combined with the current computation advancements. Here, a dataset of 406,747 unique NPs was screened against the SARS-CoV-2 main protease (M^pro^) crystal structure (6lu7) using a combination of ligand- and structural-based virtual screening. Based on 1) the predicted binding affinities of the NPs to the M^pro^, 2) the types and number of interactions with the M^pro^ amino acids that are critical for its function, and 3) the desirable pharmacokinetic properties of the NPs, we identified the top 20 candidates that could potentially inhibit the M^pro^ protease function. A total of 7 of the 20 top candidates were subjected to in vitro protease inhibition assay and 4 of them (4/7; 57%), including two beta carbolines, one N-alkyl indole, and one Benzoic acid ester, had significant inhibitory activity against M^pro^ protease. These four NPs could be developed further for the treatment of COVID-19 symptoms.

## 1. Introduction

The coronavirus disease 2019 (COVID-19) pandemic has caused serious negative social and economic impacts on the world. As of January 2023, the SARS-CoV-2 virus (the causal agent of COVID-19) has infected more than 665 million people worldwide and has resulted in 6.7 million deaths (Johns Hopkins Coronavirus Resource Center, https://coronavirus.jhu.edu/map.html, (accessed on 6 June 2021)). The uncontrolled spread of the virus contributed to the emergence of novel SARS-CoV-2 variants of concern such as the UK variant [1], Brazil variant [2], South Africa variant [3], Delta variant [4], and lastly the Omicron variant [5]. Consequently, several countries became the epicenter of the COVID-19 pandemic at different times. The development of vaccines substantially contributed to slowing the spread of the virus. Vaccination also reduced adverse outcomes such as ICU hospitalizations and deaths by 63–69% [6]. Despite the benefits of the vaccines, we still urgently need effective drugs to further reduce the number of casualties associated with SARS-CoV-2 infections in the vulnerable population.

Viruses encode several proteins that are essential to the different stages of their life cycle from infecting the host to replicating and spreading to new hosts. SARS-CoV-2 encodes 27 proteins [7] and the viral main protease (M^pro^; also called 3C-like protease; 3CL^pro^) has emerged as a potential target for antiviral drug design [8] since it is conserved in coronaviruses, plays important roles in viral gene expression and replication, and has no human homolog [9]. Antiviral drugs bind to viral proteins and disrupt the normal viral life cycle as means of protecting the host from severe and sometimes detrimental consequences. To this date, effective natural antiviral drugs are not available for the treatment of severe illnesses caused by SARS-CoV-2.

The unprecedented advancements in computational resources over the last three decades have greatly improved the efficiency of finding new drugs. With modern parallel computing, millions of compounds can be virtually screened against viral proteins in a relatively short time and ranked according to the calculated binding affinity [10]. Thus, markedly decreasing the number of potential lead molecules that need to be experimentally tested for validating their binding efficacy against the viral target protein. There are two general approaches to virtual drug screening. Ligand-based virtual screening (LBVS) is a widely used high-throughput in silico drug screening approach that makes use of the known properties of the compounds (ligands) to evaluate their suitability to bind to a receptor (such as viral protein) [11]. The two main advantages of an LBVS approach are its speed and the fact that it does not rely on the availability of the three-dimensional (3D) structure of the target, which is often a limiting factor. Thus, LBVS can be used as a first step to screen for the binding affinities of hundreds of thousands of compounds against the target and select potential candidates predicted to strongly bind to the target of interest. On the other hand, structure-based virtual screening (SBVS) depends on the 3D structure of the target molecule. The SBVS approach is computationally demanding but performs close inspection of the binding site topology of the target, such as the occurrence of cavities, clefts, sub-pockets, and electrostatic properties, to find the compounds that would most effectively bind to the target [12]. Therefore, SBVS results provide substantial validation of the compounds identified by the LBVS approach to bind the target of interest. Thus, when applied sequentially, referred to as a hybrid approach, these two approaches complement each other by reducing the time and cost of the resources needed to screen many compounds.

Given the urgency to combat the devastating COVID-19 pandemic, the most direct route to antiviral drug discovery was repurposing existing Food and Drug Administration (FDA) approved drugs against other diseases [13], mostly because starting with existing pharmacological agents that have already undergone clinical trials saved significant time [14]. Consequently, several currently available drugs have been tested for the treatment of COVID-19 but have shown little success. For instance, several drugs, such as chloroquine, lopinavir, and ritonavir, are known to inhibit the replication of other coronaviruses in vitro [15]. However, these drugs have shown no benefits when tested in hospitalized adult patients with severe COVID-19 [16]. Similarly, hydroxychloroquine, a polymerase inhibitor classically used as antimalarial medication, was not associated with a reduction in death among hospitalized patients with COVID-19 [17]. Currently, nafamostat and camostat are being evaluated in clinical trials in the treatment of COVID-19. Camostat shows inhibitory effects on the SARS-CoV-2 in TMPRSS2-expressing human cells [18] while nafamostat can block the SARS-CoV-2 fusion and significantly inhibit the cell infection of SARS-CoV-2 [19,20]. Screening a library of 10,000 compounds in preclinical stages identified seven compounds, including ebselen, which in antiviral activity assay showed the strongest antiviral effects at a concentration of 10 μM treatment in SARS-CoV-2-infected Vero cells [21]. A recent study identified the phosphoinositide 3-kinase (PI3K)/AKT signaling pathway as a common signaling pathway between cancer and COVID-19 disease [22]. Capivasertib is a potent pan-AKT kinase inhibitor drug that inhibits AKT1, AKT2 and, AKT3, and is currently being used as an oral small-molecule AKT inhibitor for drug-resistant breast cancer in clinical trials [23]. While this anticancer drug did not have a role in viral genome replication/expression, it could prevent the entry of SARS-CoV-2 into cells [22]. Thus, Capivasertib has great potential for treating cancer patients with COVID-19.

A wide range of synthetic small molecules have also been screened for drug development purposes against COVID-19. Using a computational structure-assisted drug design approach, an inhibitor named N3 was designed that showed fast in vitro inactivation of many coronavirus M^pro^s including SARS-CoV-2, by fitting inside the substrate-binding pocket [24]. In experimental studies, N3 showed strong antiviral effects at a concentration of 10 μM treatment in SARS-CoV-2-infected Vero cells [21]. Similarly, by targeting the substrate-binding pocket of SARS-CoV M^pro^, two additional inhibitors were designed and synthesized that exhibited 96–100% SARS-CoV-2 M^pro^ inhibition activity. Structural analysis showed covalent interaction between the aldehyde group of the inhibitors and the M^pro^ Cys145 residue [9]. By redesigning a previously synthesized peptidomimetic α-ketoamides developed as a broad-spectrum inhibitor of the coronavirus main proteases, ref. [25] generated compound 13b that inhibited SARS-CoV-2 replication in human Calu-3 lung cells. The COVID Moonshot non-profit and open-science consortium identified several key compounds (such as DNDI-6510) and is constantly working towards the discovery of safe, globally affordable, and easily-manufactured antiviral drugs against COVID-19 and future viral pandemics [26]. Finally, an orally bioavailable SARS-CoV-2 main protease inhibitor (PF-07321332) was developed by Pfizer showing in vitro antiviral activity with excellent off-target selectivity and in vivo safety profiles [27]. 

Synthetic drugs, while effective, are well known to occasionally cause adverse or side effects [28,29,30]. On the other hand, natural products (plant products in particular) are generally believed to be safer if identified accurately, as deaths or hospitalizations due to herbs are very rare [31]. Owing to their vast chemical diversity, natural products (NPs) offer great promise as potentially effective antiviral drugs. Baicalin and baicalein, two NPs derived from Chinese traditional medicines, exhibited potent in vitro anti-SARS-CoV-2 activities [32]. Silvestrol, an NP from *Aglaia foveolata* Pannell, is a specific inhibitor of eukaryotic translation factor eIF4A [33] and also has antiviral activity by inhibiting the replication of Ebola, Coronaviruses, hepatitis E virus, and Zika virus [34,35,36,37]. NPs of non-plant origin have also been investigated as potential M^pro^ inhibitors. Screening of a marine NP library, using both pharmacophore model and molecular docking approaches, identified 17 potential SARS-CoV-2 M^pro^ inhibitors [38]. Similarly, through computational ligand screening of 50,000 NPs from the ZINC Database [39], identified 11 NPs with the potential to effectively inhibit the SARS-CoV-2 M^pro^ function. All these potential NPs await experimental validation.

In this manuscript, we screened the binding affinity of 406,747 NPs from the most comprehensive database of NPs, called COCONUT, against the SARS-CoV-2 M^pro^ using a combination of ligand-based and structural-based hybrid virtual screening. We identified a total of 20 potential candidates with predicted inhibitory effects and provided in vitro experimental evidence of the inhibitory effects of four natural products against the SARS-CoV-2 main protease activity.

## 2. Materials and Methods

### 2.1. Datasets

An aggregated collection of NPs derived from 123 resources called MongoDB COlleCtion of Open Natural prodUcTs (COCONUT for short) [40] was used as input in the virtual screening performed in this study. This database provides information about NP structures cited in scientific literature since 2000 and contained a total of 406,747 unique molecules at the time of our study. A second database called BindingDB [41] was used for training the virtual screening engine (DeepPurpose) [42]. BindingDB is a public database that provides binding affinity information for proteins considered to be drug-targets with small molecules. It had 41,328 entries, each with a DOI, containing 2,240,573 binding data for 8503 protein targets and 971,073 small molecules at the time of our study.

### 2.2. Ligand-Based Virtual Screening

Machine learning is commonly used as a drug discovery tool for predicting protein-ligand affinities also known as drug-target interaction (DTI) [43]. A hybrid virtual screening approach was utilized in this study which included two main steps that were executed sequentially [43]. The initial step was the LBVS and was performed using the Deep Learning-based Machine learning platform called DeepPurpose [42]. DeepPurpose integrates a variety of encoding methods of drug molecules and protein amino acid sequences for DTI prediction. In addition, DeepPurpose offers a comprehensive library for DTI prediction and supports customized prediction models. To run the LBVS, the simplified molecular-input line-entry system (SMILES) notations of the 406,747 NPs derived from the COCONUT database and the amino acid sequence of the SARS-CoV-2 M^pro^ protein were encoded. The Morgan fingerprint encoder was used for the “compound encoder” while the Amino Acid Composition (AAC) was used for the “protein encoder”. The encoder process of DeepPurpose resulted in two different embeddings, namely drug and protein embeddings. These learned embeddings were concatenated and fed into an MLP-based decoder to predict the probability of binding between each NP and the target M^pro^ protein sequence. The binary classification option was selected as the classification method. The model used in DeepPurpose was trained using the BindingDB dataset described above. The BindingDB dataset was split up into training, validation, and test sets during the model training and evaluation process at a proportion of 70/10/20 training/validation/test. Of the top candidates with at least 50% binding probability predicted by LBVS, only NPs that showed no violation of Lipinski’s five rules were retained and subjected to SBVS using AutoDock Vina 1.1.2 [44].

### 2.3. Performance Measures

Receiver Operating Characteristic (ROC) and Precision-Recall curves were used to evaluate and interpret the prediction of probabilities in the binary classification system used in the LBVS process. ROC graph is a commonly used technique for visualizing, organizing and selecting classifiers based on their performance. It is a two-dimensional graph that shows the true positive rate on the *y*-axis and the false positive rate on the *x*-axis. A ROC graph depicts relative tradeoffs between benefits (true positives) and costs (false positives). Classifiers that fall on the upper left-hand side of a ROC graph are considered good with the point (0, 1) representing perfect classification that makes positive classifications only with strong evidence and makes few false positive errors [45]. The accuracy of the model was assessed by calculating the Area Under Curve (AUC) against the test datasets. The AUC represents the degree of separability. It indicates how much the model can distinguish between classes. The higher the AUC, the better the model is at predicting true positives as positives and true negatives as negatives [46].

### 2.4. Molecular Docking

The SARS-CoV-2 M^pro^ crystal structure used as the molecular docking targets were downloaded from the protein data bank (PDB ID: 6LU7). Among more than 200 Mpro protein structures, this structure was selected since it is the most commonly investigated structure in the literature. To specifically target the area harboring the active site pocket of the M^pro^ enzyme, a grid box was generated with the Cys145 amino acid residue at the center of the grid box (Table 1). To prepare the receptors, PDBQT files were created containing only the polar hydrogen atoms as well as partial charges using the python script prepare_receptor4.py (supplemental material). The missing hydrogens in the protein structures were added using the REDUCE software [47]. The ligand file was prepared in a similar way. A PDBQT file was created from a ligand molecule file using the prepare_ligand4.py (supplemental material). The processed PDBQT files were used to run Autodock Vina 1.1.2 to predict the binding affinities of multiple poses between the complex of the ligand and the target. Autodock Vina generated multiple poses for a given ligand and target complex and the best pose was selected based on the lowest binding affinity. The identified NP ligands were sorted based on their binding affinities (lowest to highest).

To validate the binding affinities obtained through Autodock Vina, molecular docking was run with two additional docking tools CB Dock (http://clab.labshare.cn/cb-dock (accessed on 6 June 2021)) and SwissDock (http://www.swissdock.ch/ (accessed on 6 June 2021)). CB Dock is a protein-ligand docking method that automatically identifies the binding sites, calculates the center and size, customizes the docking box size according to the query ligands, and then performs the molecular docking with AutoDock Vina. SwissDock is a web service to predict the molecular interactions that may occur between a target protein and a small molecule using a docking tool called EADock DSS.

### 2.5. Visual Validation of Drug-Target Interactions

The top 81 NP candidates from docking runs performed against the 6LU7 crystal structure of the SARS-CoV-2 M^pro^ were further analyzed. The docking visualization of these NP candidates was performed on PyMOL 2.5 and BIOVIA Discovery Studio Visualizer (https://discover.3ds.com/ (accessed on 12 July 2021)) to visually inspect the docking location of the NP ligand within the M^pro^’s target cavity. The types and number of bonds present between the NP ligand and the Thr24, Thr26, His41, Phe140, Asn142, Gly143, Cys145, His163, His164, Glu166, and His172 amino acid residues of each M^pro^ protein structure were observed using the BIOVIA Discovery Studio Visualizer. The given 11 amino acid residues were previously shown in the literature to play important roles in the activity of the SARS-CoV-2 M^pro^. These interactions were analyzed and weighted to further validate the efficacies of the results obtained from the molecular docking analysis from the previous step. Observations were made based on the number of hydrogen bonds and other interactions present between the NP ligands and residues within M^pro^’s target cavity. An arbitrary “Hit Score” was calculated by assigning a value of “1” if the ligand had a hydrogen bond with the 11 amino acid residues of M^pro^ and a value of “0.5” with any other types of interactions with the 11 amino acids (Table S2).

### 2.6. In Vitro Protease Inhibition Assay

The seven NPs received as powder stock were resuspended to 10 mM concentration in DMSO (Thermo Fisher Scientific, Inc., Fremont, CA, USA). The starting NP concentration of 200 µM was subjected to 10 dose IC50 assay (threefold dilution factor) according to the protocol by Reaction Biology Corp (Malvern, PA, USA) [48]. Initially, 2X enzyme or buffer as no enzyme control was delivered into the wells (Table 2). Compounds diluted in the buffer to 200 µM as the highest concentration (total 2% DMSO) or 2% DMSO as no compound control were added into the wells. After an incubation of 20 min, 2X substrate was delivered to initiate the reaction. After spin and shake, measurement by EnVision plate reader (PerkinElmer, Waltham, MA, USA) was started at room temperature, for 25 measurements with 5 min intervals (total measurement time is 2 h). Data were analyzed by taking the slope (signal/time) of the linear portion of the measurement. The slope was calculated using Excel, and curve fits were performed using Prism software v6 (GRAPH PAD software Inc., San Diego, CA, USA). GC376 inhibitor (Aobious; Cat # AOB36447) was used as a positive control. According to the RBC recommendations, NPs with IC50 values less than 2 × 10^−4^ M are considered to have a significant inhibitory effect25.

## 3. Results

**Figure 1:**
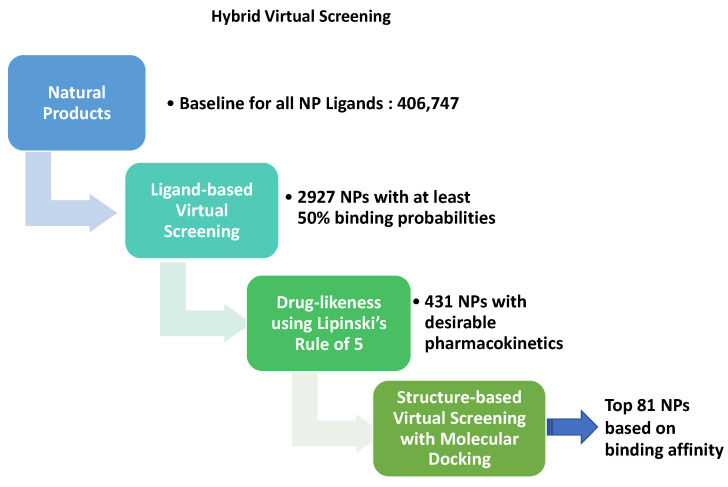
Overview of the results from LBVS and SBVS of NPs from the COCONUT database.

To predict the efficacy of NPs in potentially inhibiting the SARS-CoV-2 main protease activity, virtual screening was performed with more than 400,000 NPs from the COCONUT database using the LBVS methodology outlined in Section 2.2. The LBVS identified 2927 NPs with at least 50% predicted binding probability (Figure 1). A total of 431 NPs were selected after filtering for desirable pharmacokinetics properties (according to Lipinski’s rule of five) and were subjected to molecular docking. The top 81 NP candidates with ≤−8 kcal/mol binding affinity, based on molecular docking against the 6LU7 crystal structure of the SARS-CoV-2 M^pro^ (commonly investigated in the literature), were selected and further analyzed (Figure 1).

### 3.1. Performance of the Machine Learning Classifier

**Figure 2:**
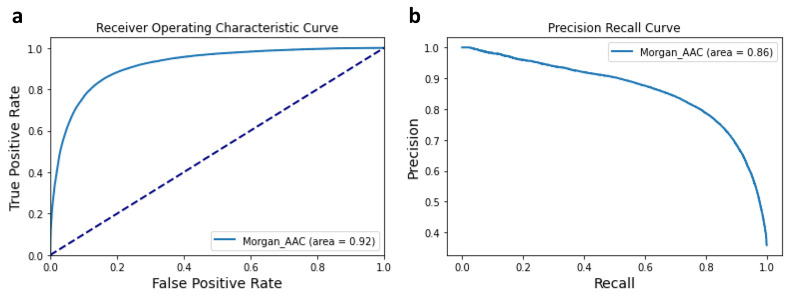
(**a**) ROC curve (Morgan + AAC) based on the test BindingDB datasets (**b**) Precision-Recall curve (Morgan + AAC) on test datasets.

The ROC-AUC measure was used to assess the performance of the training model used in this study and to calculate its probability of making a correct binary classification. With an AUC value of 0.92 (Figure 2a), the accuracy of the binary classification system is considered outstanding [49]. This result was confirmed by the precision and recall (PR) curve (Figure 2b), which shows that the model provides better precision (more true positives) at the expense of having lower recall (more misses of predicting true positives) which reduces the number of false positives.

### 3.2. Virtual Screening for NP inhibitors of M^pro^

**Figure 3:**
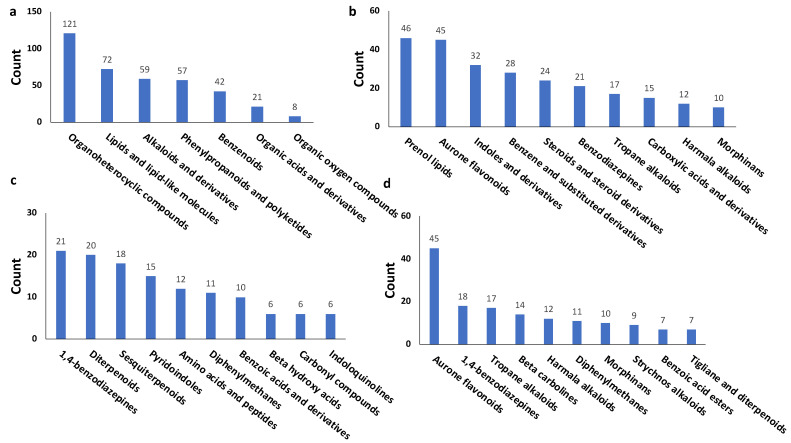
The top chemical categories represented within the 431 NP ligands identified as having potential to bind SARS-CoV-2 Mpro protein. (**a**) Chemical superclass, (**b**) Chemical class, (**c**) Chemical subclass, (**d**) Direct parent classification.

The initial LBVS with the deep learning platform called DeepPurpose identified 2927 NP ligands (0.72% of the total NPs screened) showing at least a 50% probability of being a positive ligand. These NP ligands were further filtered to retain only those NPs that do not show any violations of Lipinski’s rule of five. A total of 431 NP ligands were identified with both a high probability of binding and an increased likelihood of high oral absorption (Table S1). These NPs represented diverse compounds (Figure 3). Around a quarter of these NPs belonged to the organoheterocyclic compounds, lipids and lipid-like molecules, and alkaloids and derivatives superclass (Figure 3a; Table S1). Within the chemical class category, prenol lipids and aurone flavonoids showed the most abundant representation (Figure 3b; Table S1), while within the chemical subclass the most abundant representations were benzodiazepines and terpenoids (Figure 3c; Table S1). In terms of direct parent classification of these NPs, 12% were represented by aurone flavonoids (Figure 3d; Table S1).

The 431 NP candidates with desirable pharmacokinetics were subjected to molecular docking to observe and analyze the interaction between these NPs and the SARS-CoV-2 M^pro^ protein structure at the atomic level with the goal of identifying more potent, selective, and efficient NPs as antiviral candidates. The binding affinities of these 431 NP candidates ranged between −9.9 and −3.7 kcal/mol. A total of 81 NPs with ≤−8 kcal/mol binding affinity were manually inspected for types and number of bonds with the critical amino acid residues believed to be important for the protease function of the M^pro^ protein (Table S2). Of these 81 NPs, 83% are represented by organoheterocyclic compounds, alkaloids and derivatives, and phenylpropanoids and polyketides superclasses. Within the chemical class category, indoles and derivatives, aurone flavonoids, and benzodiazepines showed the most abundant representation (Table S3), while within the chemical subclass, the most abundant representatives were pyridoindoles and 1,4-benzodiazepines. In terms of direct parent classification of these NPs, 30% were represented by beta carbolines and aurone flavonoids (Table S3). Based on the types and number of bonds with the critical M^pro^ amino acid residues (see Section 2.5 for details), the top 20 NP candidates were selected (Table 3).

**Figure 4:**
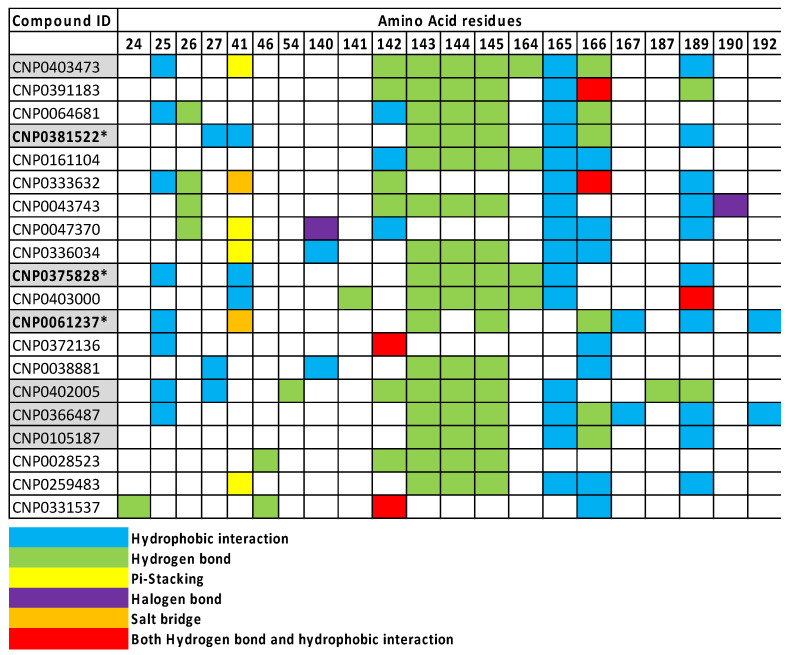
The interactions of the top 20 NP ligands with 6LU7 M^pro^ amino acid residues. The specific types of interactions between the ligands and the 21 amino acid residues of the M^pro^ are designated by different colors. The NP COCONUT ids are presented on the *y*-axis and amino acid residues of M^pro^ on the *x*-axis. The gray color indicates the seven in vitro tested compounds. COCONUT Ids in bold, gray color and with asterisk show in vitro inhibition with IC50 less than 4.52 × 10^−4^ M.

The top 20 NP ligands were 50% represented by organoheterocyclic compounds superclass, 40% by indoles and derivatives and benzodiazepines class, 30% by 1,4-benzodiazepines and pyridoindoles subclass, and 30% by 1,4-benzodiazepines and beta carbolines (Table S4). The amino acid residues 143, 144, and 145 of M^pro^ were targeted by 80% (16/20) of the top 20 NP ligands. Interestingly, all the interactions at these three amino acids were hydrogen bonds (Figure 4). Amino acid 165 had hydrophobic interactions with 75% (15/20) of the NP ligands.

### 3.3. In Vitro Protease Inhibition Assay of Selected NP Candidates

**Figure 5:**
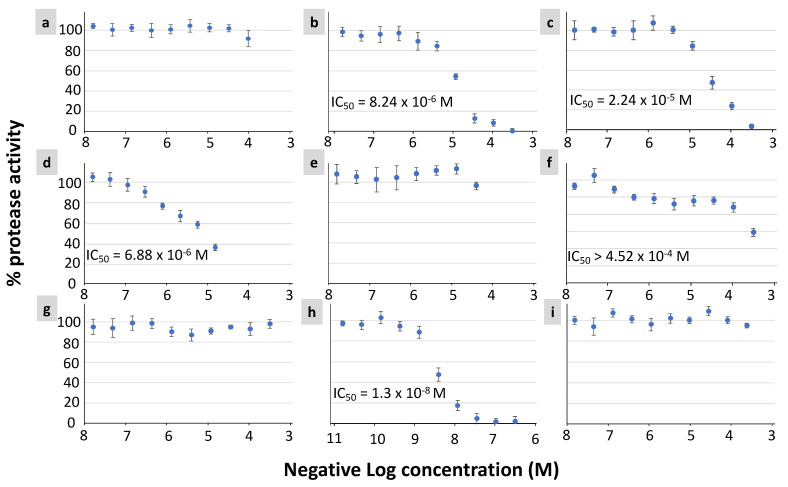
The results of in vitro protease inhibition assay of the seven NP ligands against the SARS-CoV-2 M^pro^ protein. The *y*-axis shows the percent M^pro^ protease activity. The *x*-axis shows the negative log_10_ transformed molar concentration of the NP ligands. A 10-dose three-fold serial dilution has been applied to the original 200 uM NP ligand. Some data points exhibited fluorescent backgrounds and were excluded from curve fitting. (**a**) CNP0403473, (**b**) CNP0381522, (**c**) CNP0375828, (**d**) CNP0061237, (**e**) CNP0402005, (**f**) CNP0366487, (**g**) CNP0105187, (**h**) GC376 (positive control), (**i**) DMSO (negative control). The IC50 values are provided for the four NPs and the positive control (subfigures **b**–**d**,**f**,**h**).

**Figure 6:**
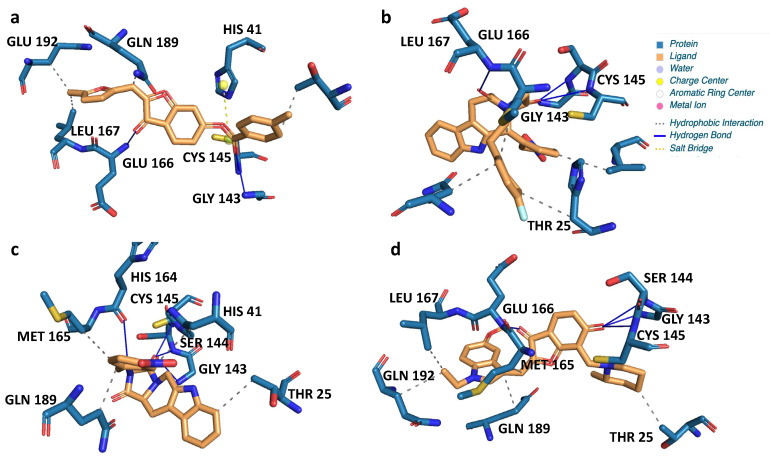
Schematics of the 3D interactions between the in vitro validated ligands and 6LU7 M^pro^ structure. (**a**) CNP0061237; (**b**) CNP0381522; (**c**) CNP0375828; (**d**) CNP0366487.

Among the top 20 NP candidates identified based on the virtual screening, 7 NPs were available for purchase (Table S5). These compounds belonged to different chemical classes (Table 4). The inhibitory activity of these seven compounds against the purified SARS-CoV-2 M^pro^ protein was assessed in vitro (Figure 5). Three NPs (CNP0061237, CNP0375828, and CNP0381522) showed significant inhibitory effects with IC50 ranging from 6.88 × 10^−6^ to 2.24 × 10^−5^ Molar (M) concentrations (Figure 5b–d). A fourth NP (CNP0366487) also showed inhibition, but to a lesser extent, with IC50 greater than 4.52 × 10^−4^ M (Figure 5f). The interactions of these NPs with the key amino acid residues of SARS-CoV-2 M^pro^ protein are presented in Table 5 and Figure 6. 

## 4. Discussion

Using a combination of ligand-based virtual screening and molecular docking against the 6lu7 protein structure of the SARS-CoV-2 main protease (M^pro^), followed by manual analysis of the ligand-target interactions, we selected 20 NPs with potential protease inhibition activity representing 0.005% of the COCONUT database with 406,747 NPs. The inhibitory activity of four NPs was experimentally confirmed in vitro. M^pro^ is a cysteine protease that is active as a homodimer [50]. The Cys145 and His41 residues present in the cleft between domains I and II in addition to the Glu166 residue are involved in the protein dimerization [51]. In line with the examination of the active site, recent studies have mentioned that the active site of this protein contains Glu166 as the most repeated and important residue, alongside Gln189, His41 and Thr190.

The molecular docking results presented in Table 3 reveal that the selected 20 NPs showed very low predicted binding affinity to the selected pocket (≤−8 kcal/mol). The 2D interaction analysis of these NPs with the 6lu7 M^pro^ structure (Table 5; Figure 4) reveals predicted interactions with the key residues in the active site (Table 4). All four NP candidates with in vitro inhibitory activity (CNP0381522, CNP0375828, CNP0061237, and CNP0366487) have predicted hydrogen bond interaction with Cys145 and hydrophobic interaction with Gln 189. Interactions with some of the other amino acid residues critical for M^pro^ function (His41 and Glu166) were also evident in these NPs with the exceptions of CNP0366487 and CNP0375828 that miss interactions with His41 and Glu166, respectively. Altogether, this suggests that one possible mechanism by which these four NPs inhibit protease activity is by inhibiting the protein dimerization of M^pro^ which is indispensable for the protease activity. Additional experiments are needed to confirm this hypothesis.

The four NPs showing in vitro inhibitory activity against M^pro^ belong to the classes of Indole and Benzene derivatives (Table 4). Due to its versatile nature with several important active positions that help bind to target proteins, many drugs with indole nuclei are currently being used in the treatment of cancer, malaria, and bacterial and viral infections [52]. In this study, we identified three Indole alkaloids (two β-carbolines and one n-alkylindole) and demonstrated their in vitro inhibitory activity against SARS-CoV-2 M^pro^. Indole alkaloids are broadly present in various plant families [53] and some interact with receptors such as opioid receptor [54]. Marine and bacterial indole alkaloids were also shown to have cytotoxic, antibacterial, antimicrobial, and antineoplastic activities [55]. β-carbolines are a specific group of biologically active and naturally occurring plant-derived alkaloids that are derivatives of indole. They are present in several plant species and exhibit a wide spectrum of biological and pharmacological effects, including antioxidant, neuroprotective, and anti-inflammatory effects [56]. The β-carbolines norharman (NH) and harman (H) are the most frequently identified carbolines. β-carbolines are found in fruits, juices, cereal products, meat, fish, and coffee which seems to be the most important food source of β-carbolines. More than 23 annomontine analogs, which represent a special class of β-carboline, were designed and docked against multiple SARS-CoV-2. Based on docking scores, the binding affinities of these annomontine derivatives were better compared to hydroxycholoquine [57]. While the potential of different β-carbolines in binding to several SARS-CoV-2 proteins has been demonstrated computationally, our results are the first to show direct in vitro experimental evidence against SARS-CoV-2 M^pro^. In the Tibetan herbal medicine *Arenaria kansuensis* extract, the relative content of the total β-carboline alkaloids was shown to be about 5% [58]. Mice with pulmonary fibrosis, a key feature of COVID-19, that were treated with this extract showed a significant increase in survival rate in a dose-dependent manner [58]. Moreover, a newly identified β-carboline alkaloid from the deep-sea fungus Trichoderma sp. MCCC 3A01244 has been shown to decrease pulmonary fibrosis by inhibiting the TGF-β/Smad signaling pathway [59]. The n-alkylindole was the third indole alkaloid natural product that inhibited the M^pro^ protease activity in our in vitro analysis. Various bisindolylmaleimide derivatives, synthesized by alkylation of the side chains of the indole nitrogen, have been shown to have pro-apoptotic activity and potential as an anti-cancer drug [60,61]. The bisindolylmaleimide GF 109203X is a potent and selective inhibitor of protein kinase C [62]. Three n-alkylated indole derivatives were shown to have different antimicrobial activity against *Staphylococcus aureus*, *Escherichia coli*, and *Candida albicans* [63]. However, n-alkylindoles have not been tested previously for their effect against SARS-CoV-2. Altogether, these promising in vitro results suggest that the indole alkaloids identified in this study could reduce SARS-CoV-2-induced life-threatening symptoms. Future in vivo and mouse model studies are needed to test this hypothesis.

Benzoic acid is the simplest aromatic carboxylic acid, with a carboxylic group directly bonded to the benzene ring. In plants, benzoic acids and their derivatives are common and widespread mediators of plant responses to biotic and abiotic stresses [64]. Many natural products derived from plant benzoic acids or containing benzoyl/benzyl moieties are also of medicinal or nutritional value to humans [65]. In the food industry, Benzoic acid and its derivatives are widely used as antibacterial and antifungal preservatives in foods [66]. Benzoic acid ester derivatives were demonstrated as potent PDE4 inhibitors for the treatment of respiratory diseases [67]. Benzoic acid esters have also been shown to have anti-microbial activity against several microbes, including mycobacteria, and represent a valuable potential for the treatment of tuberculosis [68]. Through computational analysis of the bioactive compounds of the herbal infusion “horchata” from Ecuador, Benzoic acid, 2-(ethylthio)-, ethyl ester was shown to have the lowest predicted free energy of binding to the SARS-CoV-2 M^pro^ [69]. In silico screening of several benzoic acid derivatives against the SARS-CoV-2 main protease identified 2,5-dihydroxybenzoic acid as the best potential candidate among the investigated structures [70]. Another in silico study provided evidence for (R)4-(1,5-dimethyl-3-oxo-4-hexenyl)-benzoic acid as a promising inhibitor of the spike and papain-like protease of SARS-CoV-2 [71]. According to a docking analysis with components of Egyptian propolis or bee glue, a resinous material produced by bees to protect their hives, benzoic acid revealed the lowest ICM scores with four hydrogen bonds with LYS110 and THR111. In vitro M^pro^ protease inhibition assay showed that propolis extract possesses a good inhibitory effect against SARS-CoV-2 M^pro^ (IC50 = 2.452 ± 0.11 µg/mL) [72]. In our study, we provide direct in vitro experimental evidence for a Benzoic acid ester inhibiting the protease activity of SARS-CoV-2 M^pro^.

## 5. Conclusions

In conclusion, the virtual screening approach used in this study was successful in identifying a high percentage (57%) of true positive candidates, at least based on in vitro experimental validation, from a database with more than 400,000 natural products. In the future, the same approach could be applied to other natural product libraries to utilize natural products as an alternative to synthetic compounds. The present finding, together with other research findings in the literature, suggest that natural products hold promise to find out novel cures for devastating diseases affecting humans. The natural products identified in this study will have to be tested in animal systems in vivo to further validate their inhibitory effect against the SARS-CoV-2 M^pro^ in a more complex and real-life setting before proceeding to clinical studies.

## Figures and Tables

**Table 1:** The coordinates and the size of the grid box on 6LU7 crystal structure.

Protein Structure	Center			Size		
	X	Y	Z	X	Y	Z
6LU7	−14.607	19.162	64.101	25	25	25

**Table 2:** Components of the assay.

Enzyme	SARS-CoV-2 Mpro
Enzyme in rxn (nM)	12
Substrate	NH2-C(EDANS)VNSTQSGLRK(DABCYL)M-CO2H
Substrate in rxn (µM)	5
Excitation/Emission	340/490
Mpro buffer	50 mM Tris pH 7.3, 1 mM EDTA, 1 mM DTT, 0.005% Triton X-100

**Table 3:** The selected top 20 NP ligands against 6LU7 M^pro^ protein structure. The last three columns show binding affinities based on Autodock Vina, SwissDock, and CBDock docking software.

			Binding Affinity (kcal/mol)		
COCONUT Id	Name	Direct Parent/Class	Autodoc Vina	SwissDock	CB Dock
CNP0403473	N-[(2H-1,3-benzodioxol-5-yl)methyl]-2-(2,5-dioxo-2,3,4,5-tetrahydro-1H-1,4-benzodiazepin-3-yl)acetamide	1,4-benzodiazepines	−8.1	−7.65	−8.6
CNP0391183	5-bromo-N-(5-chloro-2-methoxyphenyl)-5′-(1-hydroxyethyl)-2-oxo-1,2-dihydrospiro[indole-3,2′-pyrrolidine]-3′-carboxamide	Indoles	−8.7	−7.94	−7.8
CNP0064681	4′-(methoxycarbonyl)-1′-methyl-2-oxo-1,2,4′a,5′,5′a,7′,8′,9′,10′,10′a-decahydro-1′H-spiro[indole-3,6′-pyrano [3,4-f]indolizin]-9′-ium	Indolizidines	−8.0	−7.97	−7.5
CNP0381522	(8R)-6-[(E)-[(4-nitrophenyl)methylidene]amino]-3,6,17-triazatetracyclo [8.7.0.0^3^,^8^.0^11^,^16^]heptadeca-1(10),11,13,15-tetraene-4,7-dione	NA	−8.9	NA	−9
CNP0161104	3-[(2-hydroxy-2,2-diphenylacetyl)oxy]-1,1-dimethylpiperidin-1-ium	Diphenylmethanes	−8.1	NA	−7.9
CNP0333632	11-(1-hydroxy-4-methylpentyl)-4-(3-methoxy-4-methyl-5-oxo-2,5-dihydrofuran-2-ylidene)-3-methyl-5-oxa-10-azatetracyclo [6.6.2.0^1^,^10^.0^2^,^6^]hexadeca-6,15-dien-10-ium	Azaspirodecane	−8.0	NA	−7.7
CNP0043743	N-[13-(4-chlorophenyl)-2,8-dioxo-3,9-diazatricyclo [8.4.0.0^3^,^7^]tetradeca-1(14),10,12-trien-5-yl]methanesulfonamide	Benzodiazepines	−8.1	−8.12	−8
CNP0047370	4-[bis(4-fluorophenyl)methylidene]-1-(2-{7-methyl-5-oxo-5H-[1,3]thiazolo [3,2-a]pyrimidin-6-yl}ethyl)piperidin-1-ium	Diphenylmethanes	−8.3	−8.03	−8.3
CNP0336034	4-chloro-2-[4-(2,3-dihydro-1,4-benzodioxin-6-yl)-1,2-oxazol-5-yl]phenol	Benzodioxanes	−8.2	−7.81	−7.8
CNP0375828	(12aS)-2-{[(E)-(4-nitrophenyl)methylidene]amino}-2,3,6,7,12,12a-hexahydropyrazino [1′,2′:1,6]pyrido [3,4-b]indole-1,4-dione	NA	−9.3	NA	−8.2
CNP0403000	3-(2,5-dioxo-2,3,4,5-tetrahydro-1H-1,4-benzodiazepin-3-yl)-N-[(1-methyl-1H-1,3-benzodiazol-2-yl)methyl]propanamide	Benzodiazepines	−8.7	−8.84	−8.7
CNP0061237	2-[(5-methylfuran-2-yl)methylidene]-3-oxo-2,3-dihydro-1-benzofuran-6-yl 4-methylbenzoate	Benzene and derivatives	−8.2	−8.33	−8
CNP0372136	17-[(4-nitrophenyl)methyl]-9-oxo-12-oxa-8,17-diazaheptacyclo [15.5.2.0^1^,^18^.0^2^,^7^.0^8^,^22^.0^11^,^21^.0^15^,^20^]tetracosa-2,4,6,14-tetraen-17-ium	Strychnos alkaloids	−8.4	NA	−8.9
CNP0038881	3-[(2-hydroxy-2,2-diphenylacetyl)oxy]-8-methyl-8-azabicyclo [3.2.1]octan-8-ium	Diphenylmethanes	−8.0	−7.53	−7.6
CNP0402005	3-(2,5-dioxo-2,3,4,5-tetrahydro-1H-1,4-benzodiazepin-3-yl)-N-(4-oxo-3,4-dihydroquinazolin-6-yl)propanamide	Diazanaphthalenes	−8.4	−8.58	−8.9
CNP0366487	1-({2-[(1-ethyl-5-methoxy-1H-indol-3-yl)methylidene]-6-oxido-3-oxo-2,3-dihydro-1-benzofuran-7-yl}methyl)-2-methylpiperidin-1-ium	Indoles and derivatives	−8.2	NA	−8.2
CNP0105187	2-[(4-fluorophenyl)methylidene]-3-oxo-2,3-dihydro-1-benzofuran-6-yl morpholine-4-carboxylate	Aurone flavonoids	−8	−8.56	−8.2
CNP0028523	NA	NA	−8	−9.51	−7.4
CNP0259483	6-cyclopentyl-2-(3-nitrophenyl)-3,6,17-triazatetracyclo [8.7.0.0^3^,^8^.0^11^,^16^]heptadeca-1(10),11,13,15-tetraene-4,7-dione	NA	−9.9	NA	−10.1
CNP0331537	Capsimycin B	Macrolactams	−8.3	−7.02	−8.9

**Table 4:** Classification of the seven NPs used for in vitro M^pro^ inhibition assay. COCONUT Ids in bold, gray color and with asterisk show in vitro inhibition.

COCONUT Id	Chemical SuperClass	Chemical Class	Chemical SubClass	DirectParent Classification
CNP0403473	Organoheterocyclic compounds	Benzodiazepines	1,4-benzodiazepines	1,4-benzodiazepines
CNP0381522 *	Organoheterocyclic compounds	Indoles and derivatives	Pyridoindoles	Beta carbolines
CNP0375828 *	Organoheterocyclic compounds	Indoles and derivatives	Pyridoindoles	Beta carbolines
CNP0061237 *	Benzenoids	Benzene and derivatives	Benzoic acids and derivatives	Benzoic acid esters
CNP0402005	Organoheterocyclic compounds	Diazanaphthalenes	Benzodiazines	Quinazolinamines
CNP0366487 *	Organoheterocyclic compounds	Indoles and derivatives	N-alkylindoles	N-alkylindoles
CNP0105187	Phenylpropanoids and polyketides	Aurone flavonoids	Unknown	Aurone flavonoids

**Table 5:** Schematics of the 2D interactions between the four in vitro validated ligands and 6LU7 M^pro^ structure.

Name	2D Structure	2D Interaction with M^pro^ Residues
CNP0381522	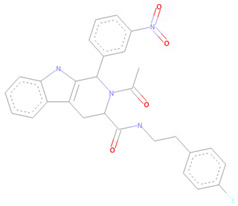	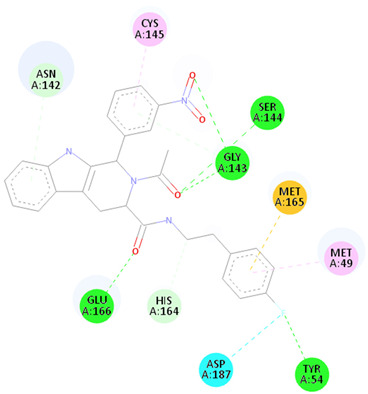
CNP0375828	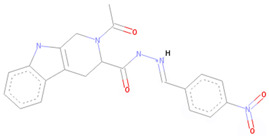	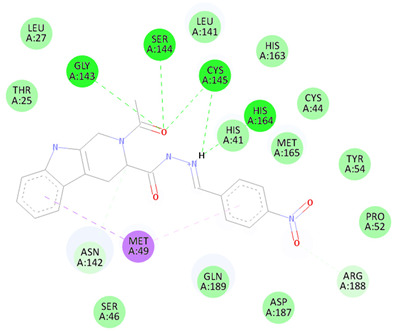
CNP0061237	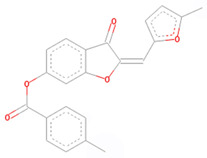	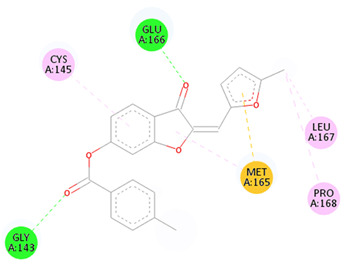
CNP0366487	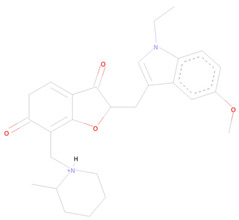	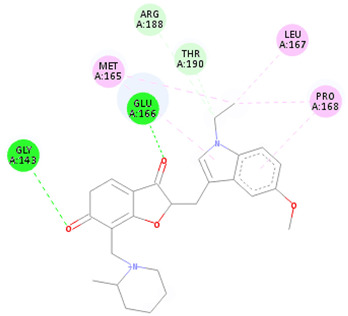
Key for 2D Interaction Maps	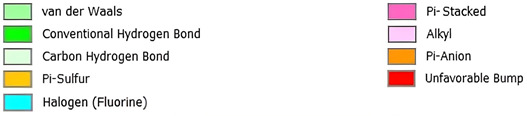	

## Data Availability

The data is provided in the Supplemental Materials.

## Supplementary Materials

The following supporting information can be downloaded at: https://www.mdpi.com/article/10.3390/biology12040519/s1, Table S1: The classification of the 431 NP ligands identified with both high probability of binding and an increased likelihood of high oral absorption. Table S2: The top 81 NPs with ≤−8 kcal/mol binding affinity. Table S3: The classification of the top 81 NPs. Table S4: Chemcial classification of the top 20 NPs. Table S5: Information about the seven compounds tested in vitro.
